# 11 A Twenty-one Year Review of Medical Malpractice Cases in Burn Care

**DOI:** 10.1093/jbcr/irac012.015

**Published:** 2022-03-23

**Authors:** Christopher H Pham, Justin Dang, Melissa Huang, Zachary J Collier, Justin Gillenwater, Warren L Garner, Richard Kelly

**Affiliations:** University of Southern California, Cypress, California; Sidney Kimmel Medical College at Thomas Jefferson University, Costa Mesa, California; University of California, Irvine, Irvine, California; Keck School of Medicine, University of Southern California, Los Angeles, California; USC/LAC+USC Medical Center, Los Angeles, California; USC, Los Angeles, California; University of California, Irvine, Irvine, California

## Abstract

**Introduction:**

Traumatic injuries are a common source of medical litigation but no prior studies have reported on the trends of medical malpractice in burn care. As a result, it is unclear what factors increase the likelihood that a medical professional caring for a burn patient will be named in a lawsuit. By understanding what drives patient discontent and the motivations behind their malpractice claims, burn care practitioners can better care for patients and decrease their own risk of involvement in medical litigation.

**Methods:**

The Westlaw legal research database contains state and federal court documents from over 40,000 databases across the United States and was queried for all cases of burn injuries and medical malpractice from 2000 to 2021. All mechanisms-of-injury (e.g. flame, scald, electrical, chemical, Stevens-Johnson, etc.) were included. Case information including injury circumstances, plaintiff/defendant occupation, defendant burn care experience, and burn demographics (TBSA, mechanism) were recorded when available. The primary outcome was the case ruling (plaintiff or defendant), and settlement amounts were recorded when available.

**Results:**

Forty of the 1,222 identified cases fit inclusion criteria. Twenty-seven percent (11/40) of cases involved treatment at a burn center and 82% of plaintiffs were men. The two most common mechanisms-of-injury were scald (38%) and flame burns (18%). The court ruled in favor of the plaintiff in 10% (4/40) of cases. When the court/jury ruled in favor of the plaintiff, the settlement amount ranged from $25,000 to $20,000,000. The most frequently sued medical specialty was Family Medicine (35%) and mid-level practitioners like physician assistants and nurse practitioners (35%). Physicians with a burn fellowship were named in only 5% (2/40) of cases. The most common claims were for burn depth misdiagnoses (7/40), deliberate indifference/treatments below standard-of-care (7/40), and delayed referrals to a burn specialist (6/40). All cases met American Burn Association transfer criteria. Patient mortality was the reason for litigation in 10% (4/40) of cases.

**Conclusions:**

This study showed that most burn-specialized practitioners are not the subject of litigation. In fact, many patients filed malpractice claims because they were not referred to burn specialists. Based on case text analysis, all of the patients’ injuries fit American Burn Association transfer criteria and some lawsuits may have been avoidable.

